# Supplementary material for: Sperm induction of somatic cell-cell fusion as a novel functional test
Source: eLife. 2024 Jan 24;13:e94228. doi: 10.7554/eLife.94228 (PMC10883674; doi:10.7554/eLife.94228)
Supplement: Source data 1. [file elife-94228-data1.docx]

Supplementary file 1: Raw data from correlation assays

|  | | |  | **Acrosome Reaction** | **IVF** | | | | | **Multinucleation** | | | | |
| --- | --- | --- | --- | --- | --- | --- | --- | --- | --- | --- | --- | --- | --- | --- |
| **Male** | | | **Total sperm** | **n= 1,000** | **COCs** | | **ZP-free** | | | **Control** | | **SPICER (with sperm)** | | **Fold increase** |
| **#** | **Age (months)** | **Genetic background** | **x10^6** | **% of acrosome reaction** | **Fertilized eggs/total** | **% of ferilization** | **Fertilized eggs/total (polyspermy)** | **% of fertilization** | **# of sperm/egg** | **Count** | **%** | **Count** | **%** |  |
| 1 | 2.97 | Hybrid FVB/129sv/CF1 | 15.2 | 24.8 | 18/18 | 100.0 | 16/18 | 88.89 | 0.89 | 80/925 | 8.65 | 299/920 | 32.50 | 3.76 |
| 2 | 4.37 | Hybrid B6D2 | 5.2 | 20.5 | 0/14 | 0.0 | 12/20 | 60.0 | 0.60 |  |  | 203/968 | 20.97 | 2.42 |
| 3 | 3.37 | Hybrid B6D2 | 6.5 | 17.8 | 12/15 | 80.0 | 12/12 (2 w/2 sperm) | 100.0 | 1.17 | 58/838 | 6.92 | 268/992 | 27.02 | 3.90 |
| 4 | 5.00 | Hybrid B6D2 | 8.3 | 38.1 | 0/12 | 0.0 | 0/14 | 0.0 | 0.00 |  |  | 100/842 | 11.88 | 1.72 |
| 5 | 3.47 | Hybrid FVB/129sv/CF1 | 14.4 | 21.8 | 22/22 | 100.0 | 19/19 (4 w/2 sperm and 3 w/3 sperm) | 100.0 | 1.53 | 72/1066 | 6.75 | 264/984 | 26.83 | 3.97 |
| 6 | 3.63 | Hybrid B6D2 | 7.5 | 16.8 | 14/19 | 73.7 | 16/16 (6 w/2 sperm) | 100.0 | 1.38 |  |  | 154/828 | 18.60 | 2.76 |
| 7 | 4.17 | Hybrid FVB/129sv/CF1 | 10.1 | 36.6 | 13/13 | 100.0 | 19/21 (6 w/2 sperm and 2 w/3 sperm) | 90.5 | 1.38 | 36/917 | 3.93 | 202/968 | 20.87 | 5.31 |
| 8 | 3.50 | Hybrid B6D2 | 5.7 | 18.5 | 0/12 | 0.0 | 17/25 (3 w/2 sperm) | 68.0 | 0.80 |  |  | 197/845 | 12.66 | 3.22 |
| 9 | 2.47 | Hybrid B6D2 | 10.2 | 44.0 | 18/18 | 100.0 | 13/13 (3 w/2) | 100.0 | 1.23 | 48/952 | 4.70 | 206/894 | 23.04 | 4.90 |
| 10 | 2.47 | Hybrid B6D2 | 3.29 | 45.5 | 0/14 | 0.0 | 1/11 | 9.1 | 0.09 |  |  | 154/958 | 16.08 | 3.42 |
