# Supplementary figures and images for: Sperm induction of somatic cell-cell fusion as a novel functional test
Source: eLife. 2024 Jan 24;13:e94228. doi: 10.7554/eLife.94228 (PMC10883674; doi:10.7554/eLife.94228)

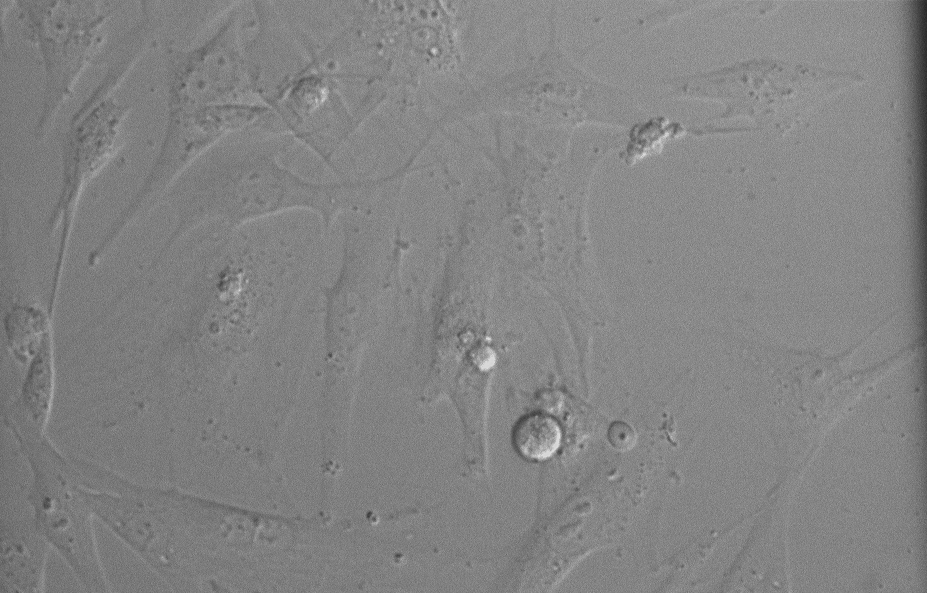

Supplement: Source data 3. [file elife-94228-data3.zip › Raw Images/Figure 1/Panel E and Movie 1/Stack DIC.tif]

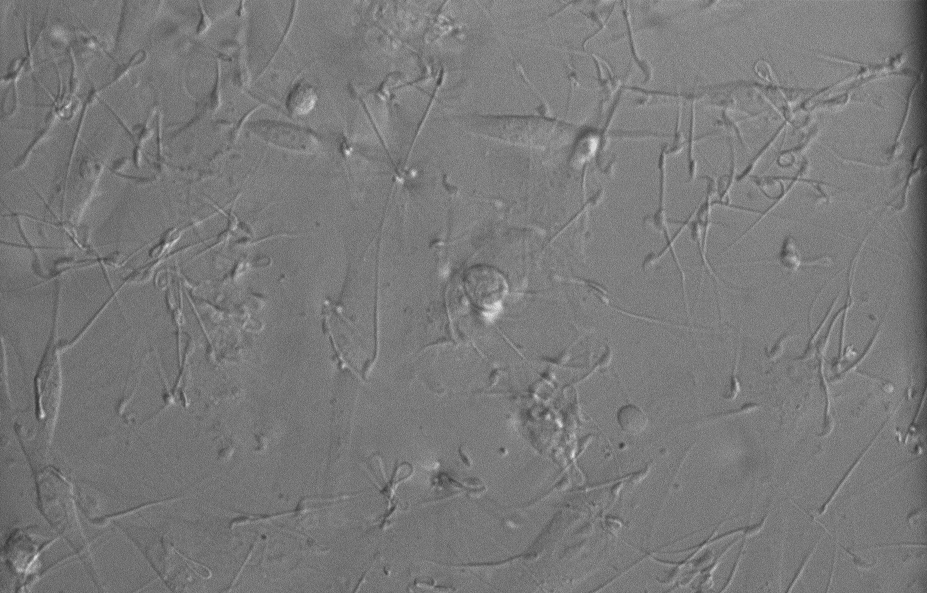

Supplement: Source data 3. [file elife-94228-data3.zip › Raw Images/Figure 1/Panel E and Movie 1/DIC/DIC_s13_t20.TIF]

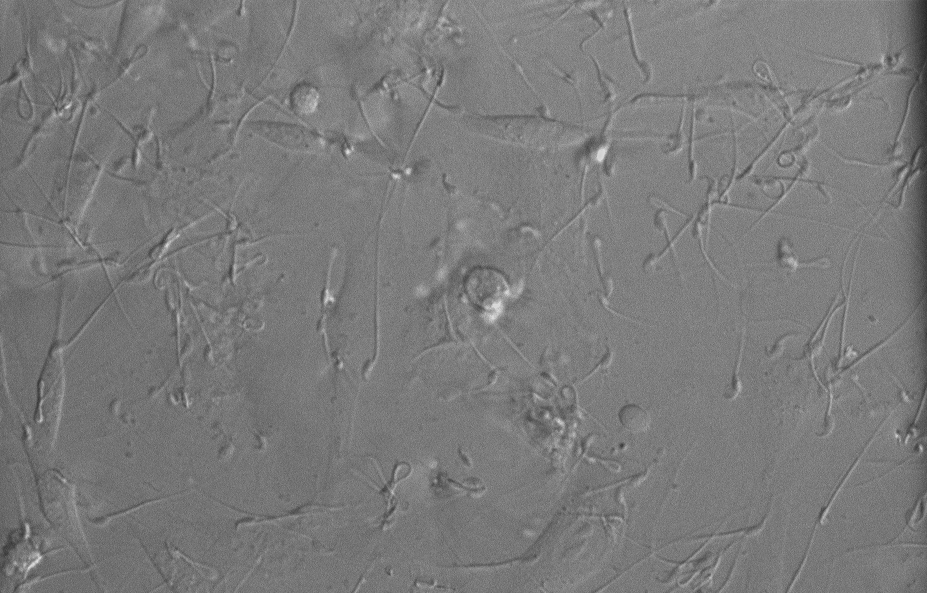

Supplement: Source data 3. [file elife-94228-data3.zip › Raw Images/Figure 1/Panel E and Movie 1/DIC/DIC_s13_t21.TIF]

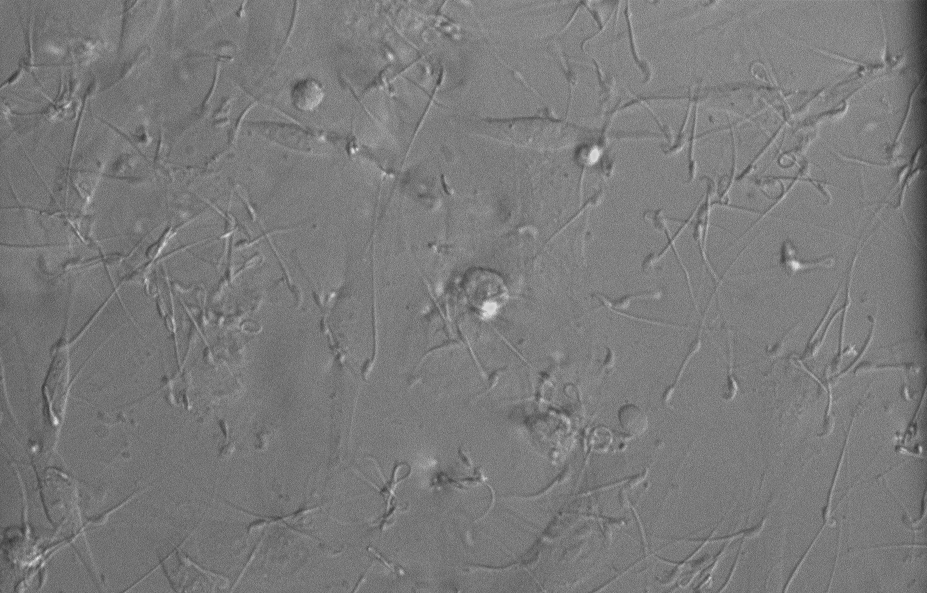

Supplement: Source data 3. [file elife-94228-data3.zip › Raw Images/Figure 1/Panel E and Movie 1/DIC/DIC_s13_t23.TIF]

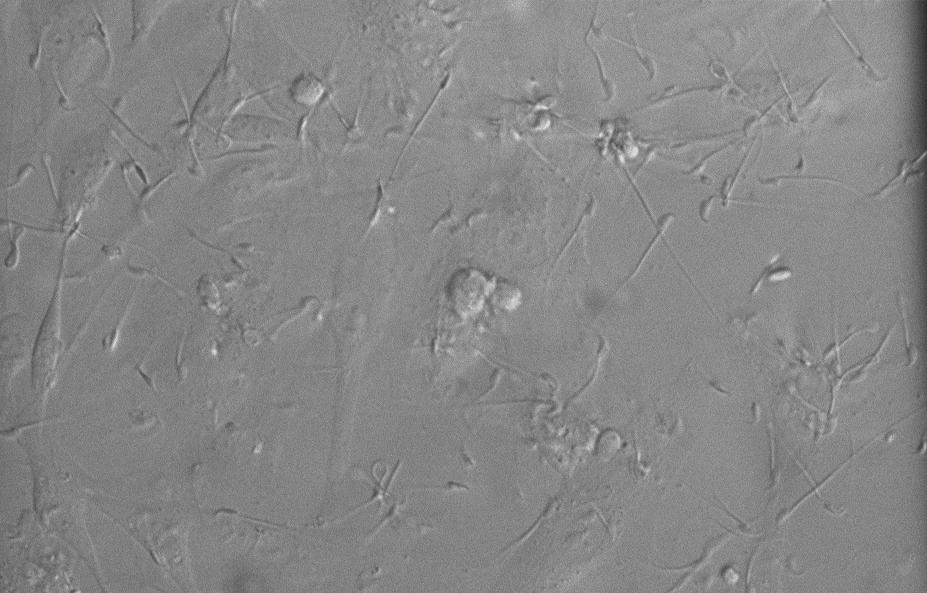

Supplement: Source data 3. [file elife-94228-data3.zip › Raw Images/Figure 1/Panel E and Movie 1/DIC/DIC_s13_t9.TIF]

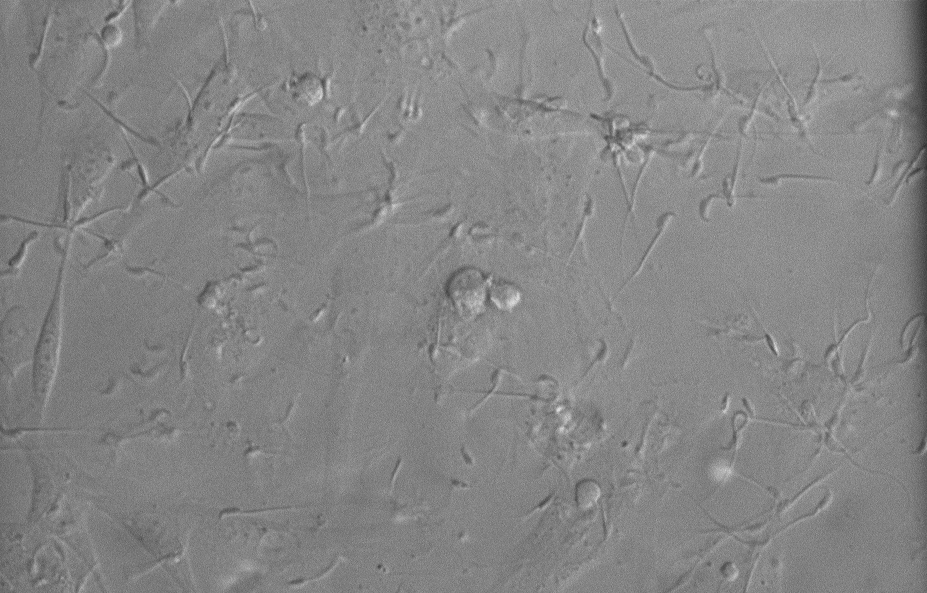

Supplement: Source data 3. [file elife-94228-data3.zip › Raw Images/Figure 1/Panel E and Movie 1/DIC/DIC_s13_t8.TIF]

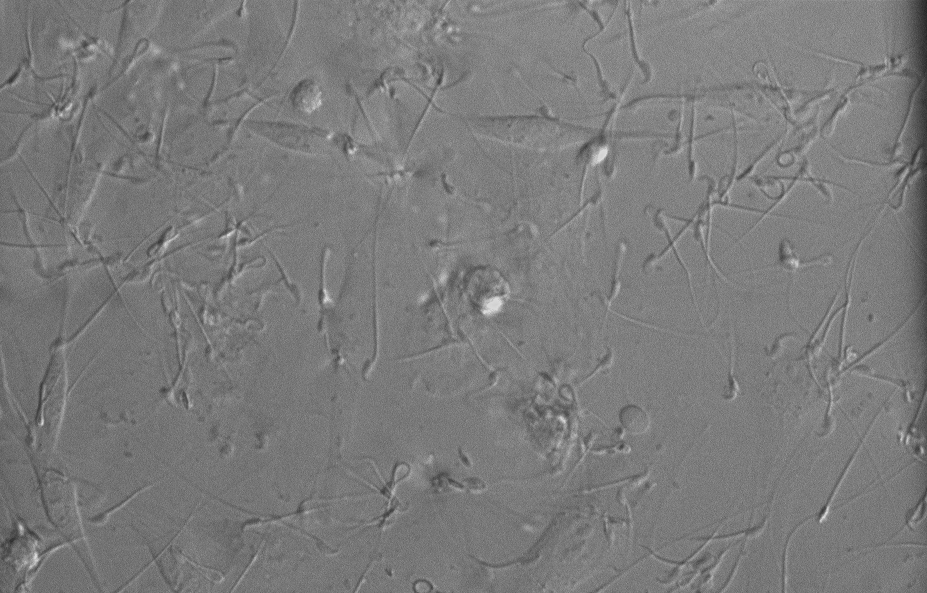

Supplement: Source data 3. [file elife-94228-data3.zip › Raw Images/Figure 1/Panel E and Movie 1/DIC/DIC_s13_t22.TIF]

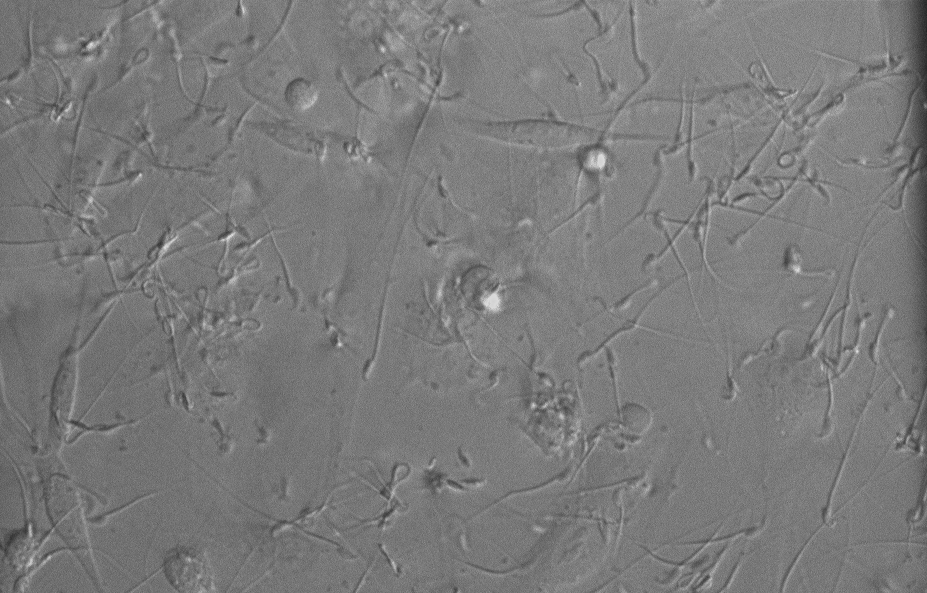

Supplement: Source data 3. [file elife-94228-data3.zip › Raw Images/Figure 1/Panel E and Movie 1/DIC/DIC_s13_t26.TIF]

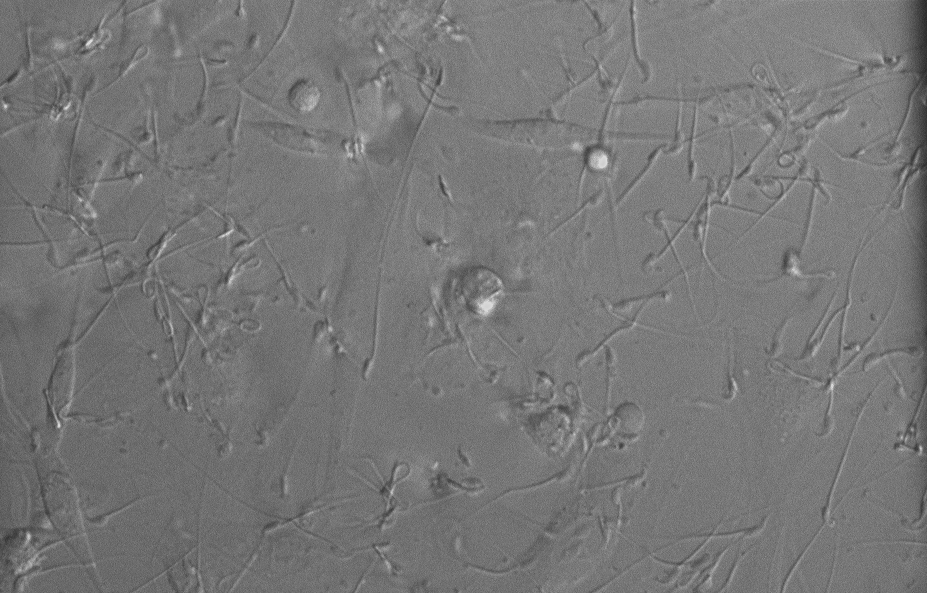

Supplement: Source data 3. [file elife-94228-data3.zip › Raw Images/Figure 1/Panel E and Movie 1/DIC/DIC_s13_t25.TIF]

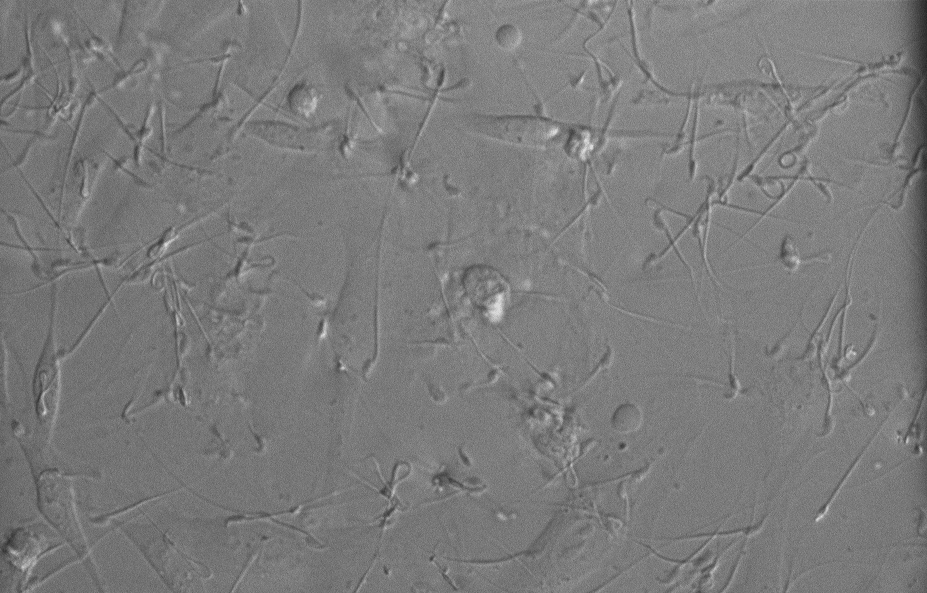

Supplement: Source data 3. [file elife-94228-data3.zip › Raw Images/Figure 1/Panel E and Movie 1/DIC/DIC_s13_t19.TIF]

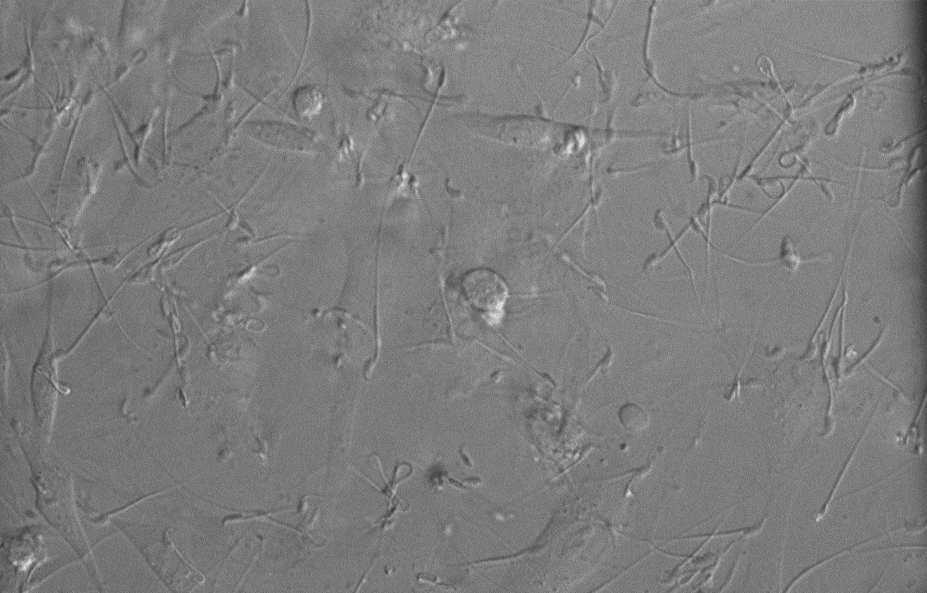

Supplement: Source data 3. [file elife-94228-data3.zip › Raw Images/Figure 1/Panel E and Movie 1/DIC/DIC_s13_t18.TIF]

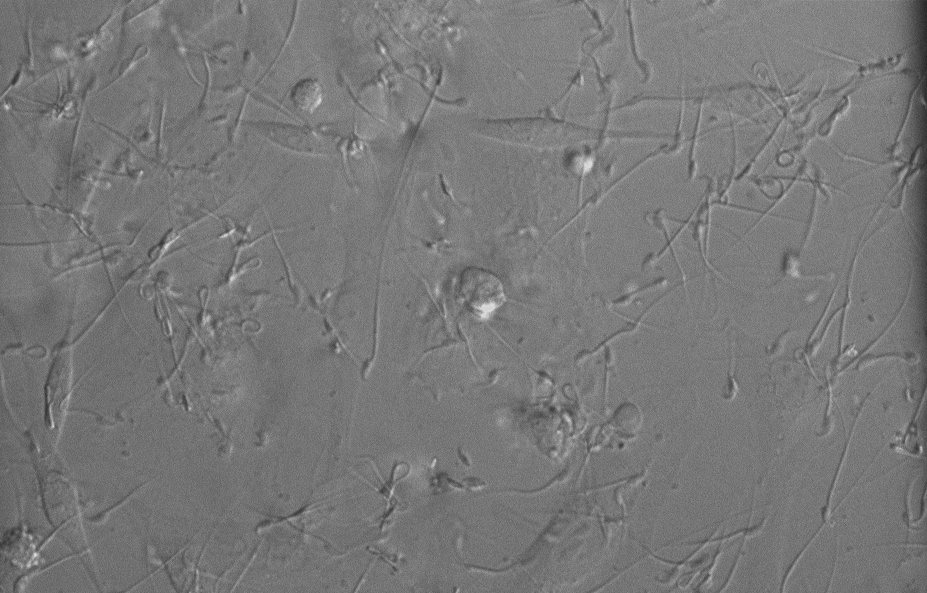

Supplement: Source data 3. [file elife-94228-data3.zip › Raw Images/Figure 1/Panel E and Movie 1/DIC/DIC_s13_t24.TIF]

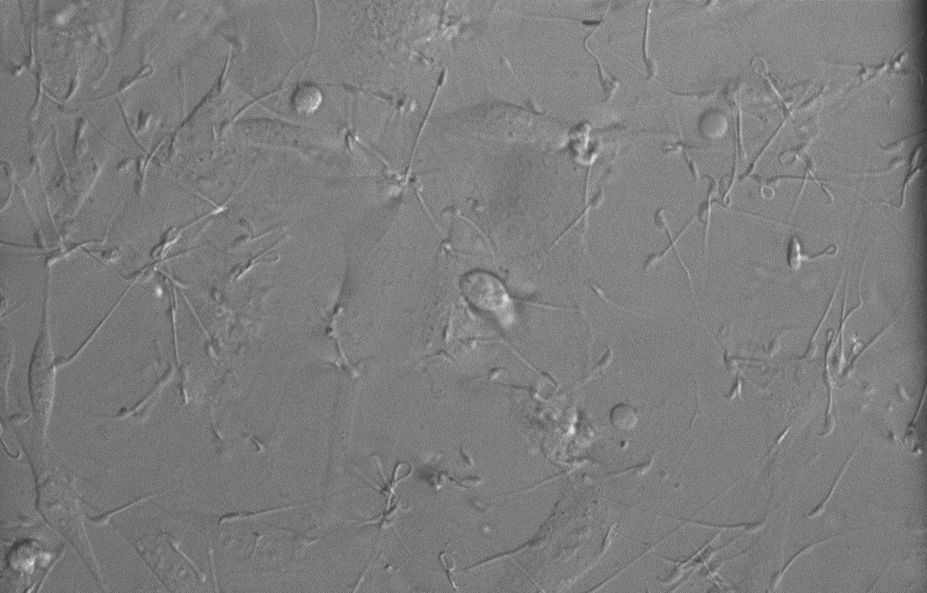

Supplement: Source data 3. [file elife-94228-data3.zip › Raw Images/Figure 1/Panel E and Movie 1/DIC/DIC_s13_t15.TIF]

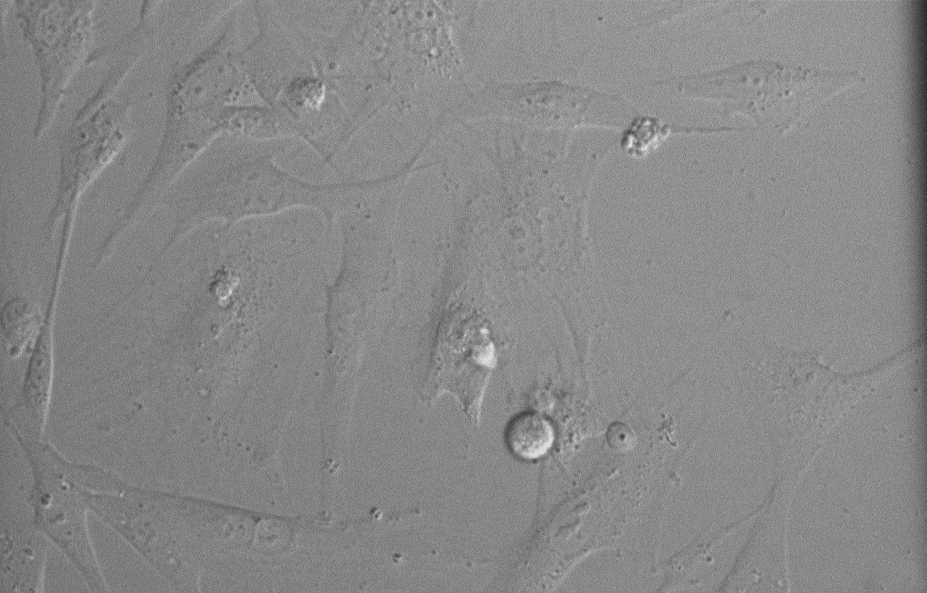

Supplement: Source data 3. [file elife-94228-data3.zip › Raw Images/Figure 1/Panel E and Movie 1/DIC/DIC_s13_t3.TIF]

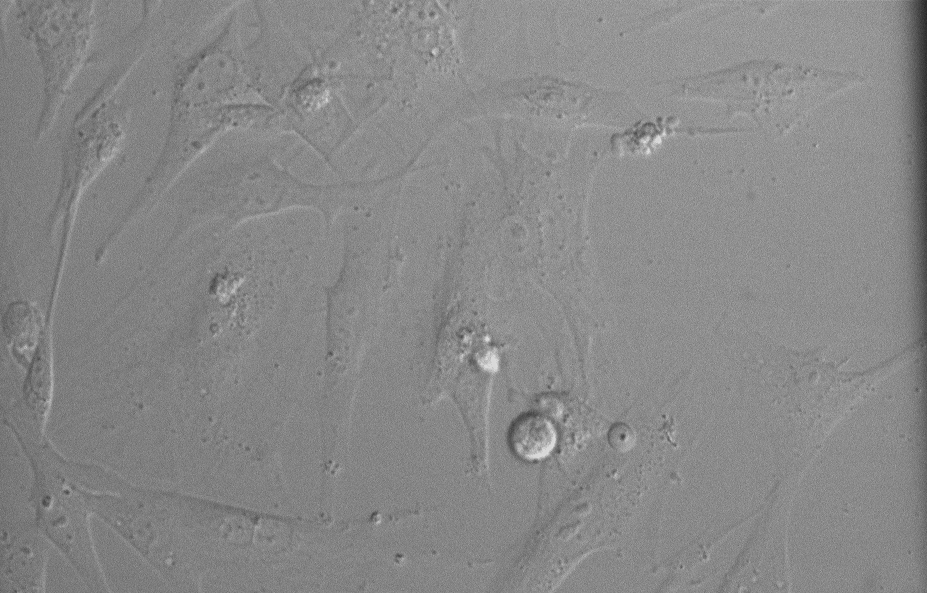

Supplement: Source data 3. [file elife-94228-data3.zip › Raw Images/Figure 1/Panel E and Movie 1/DIC/DIC_s13_t2.TIF]

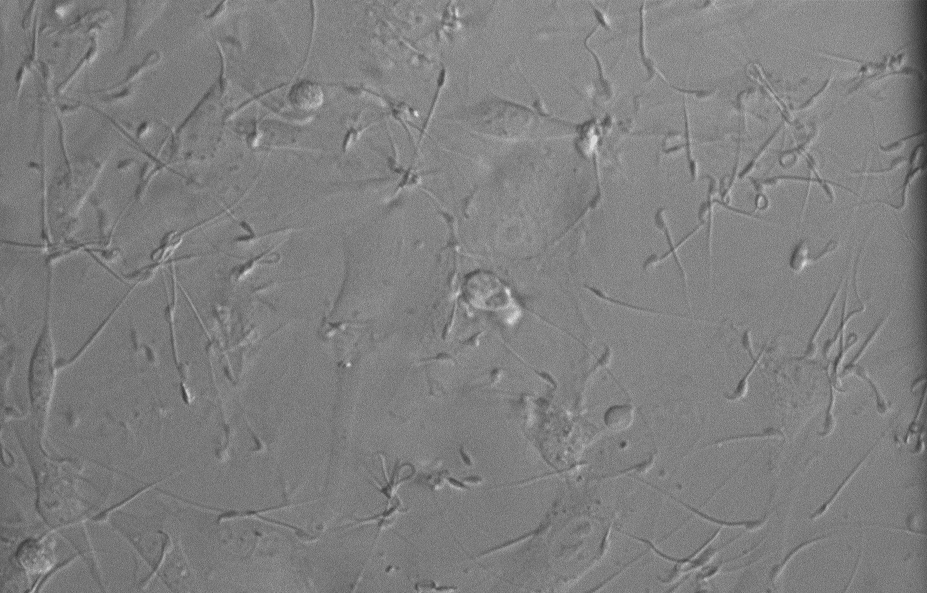

Supplement: Source data 3. [file elife-94228-data3.zip › Raw Images/Figure 1/Panel E and Movie 1/DIC/DIC_s13_t14.TIF]

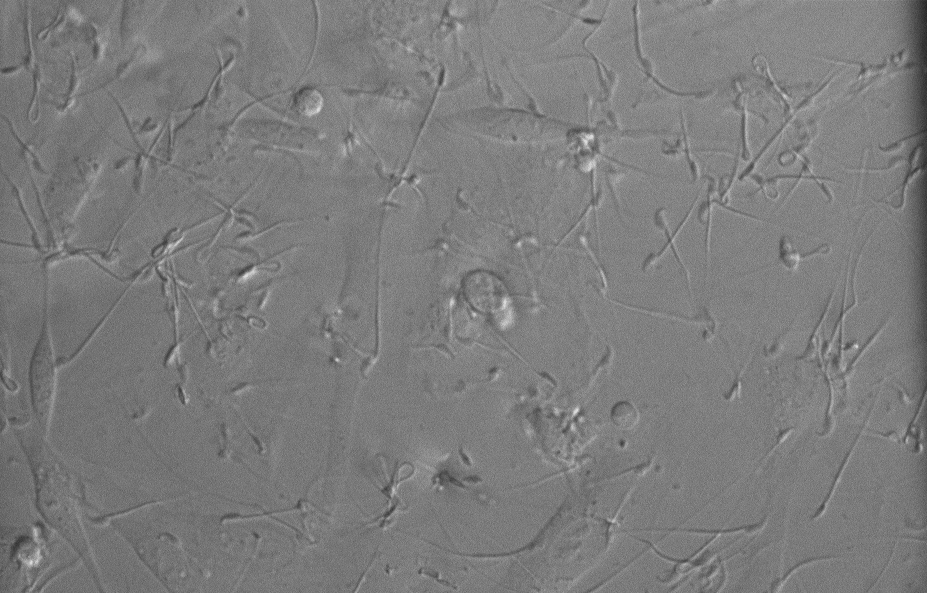

Supplement: Source data 3. [file elife-94228-data3.zip › Raw Images/Figure 1/Panel E and Movie 1/DIC/DIC_s13_t16.TIF]

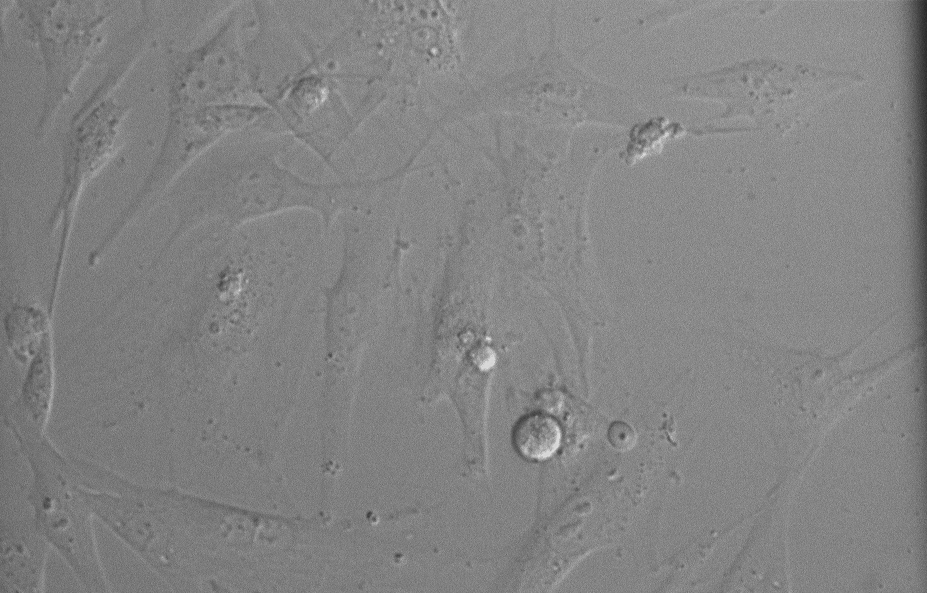

Supplement: Source data 3. [file elife-94228-data3.zip › Raw Images/Figure 1/Panel E and Movie 1/DIC/DIC_s13_t1.TIF]

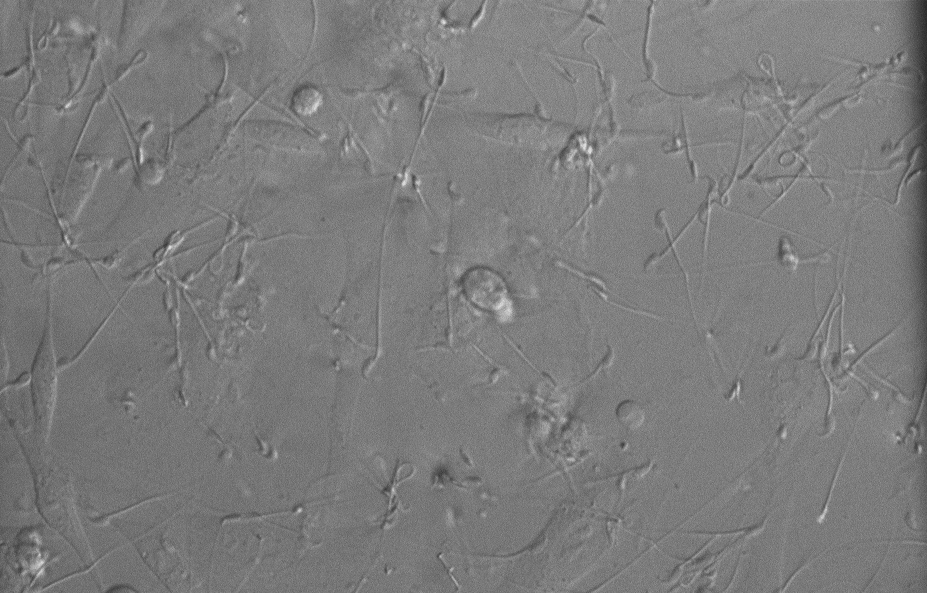

Supplement: Source data 3. [file elife-94228-data3.zip › Raw Images/Figure 1/Panel E and Movie 1/DIC/DIC_s13_t17.TIF]

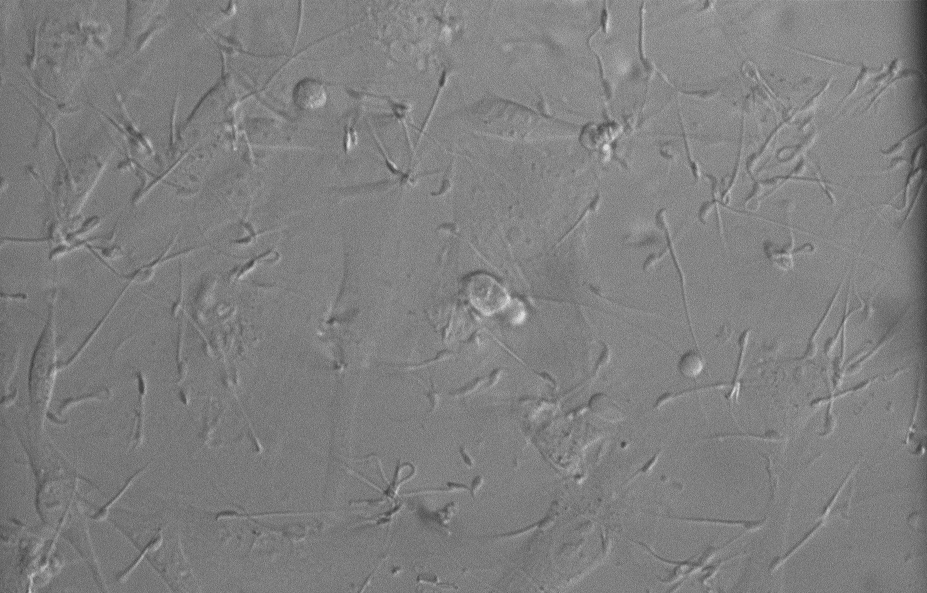

Supplement: Source data 3. [file elife-94228-data3.zip › Raw Images/Figure 1/Panel E and Movie 1/DIC/DIC_s13_t13.TIF]

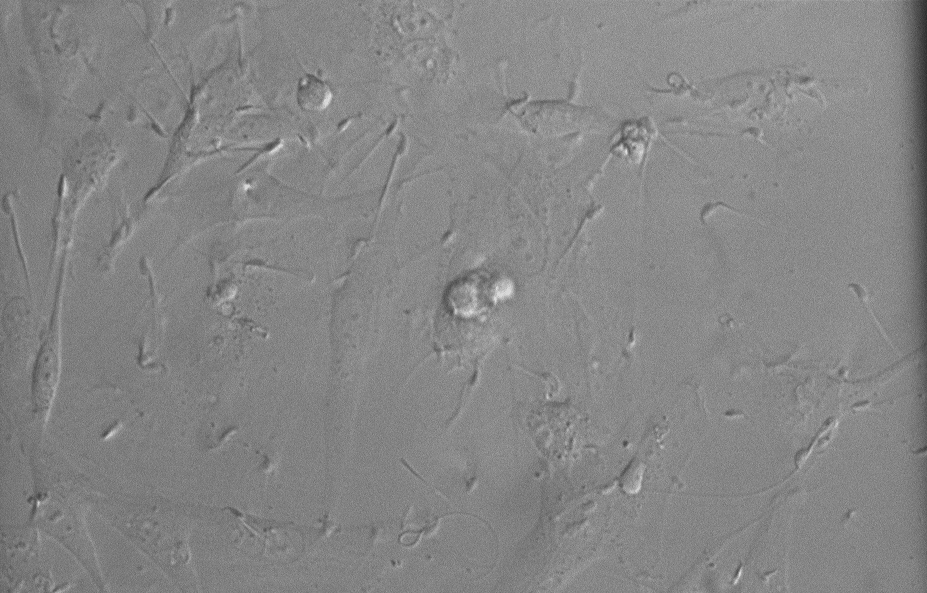

Supplement: Source data 3. [file elife-94228-data3.zip › Raw Images/Figure 1/Panel E and Movie 1/DIC/DIC_s13_t5.TIF]

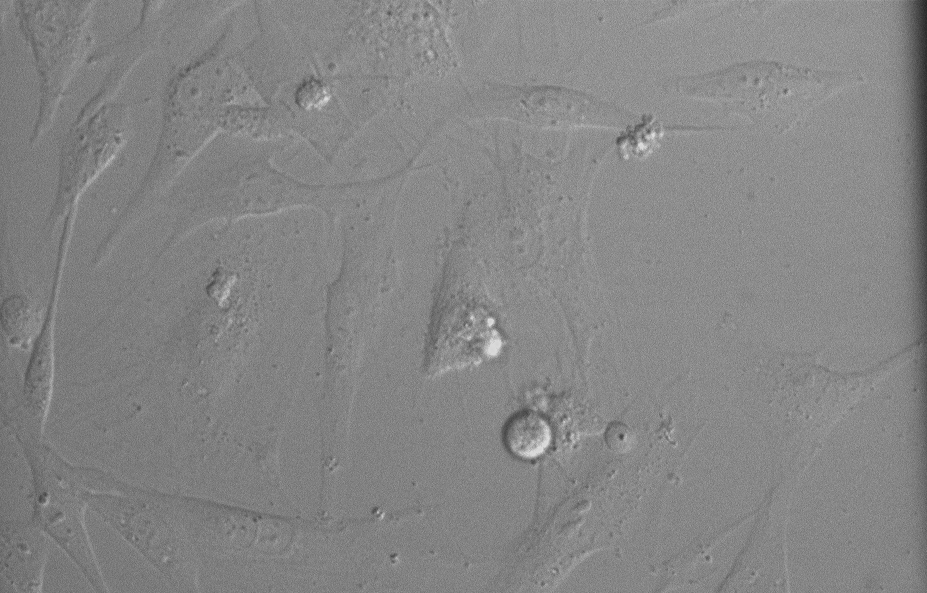

Supplement: Source data 3. [file elife-94228-data3.zip › Raw Images/Figure 1/Panel E and Movie 1/DIC/DIC_s13_t4.TIF]

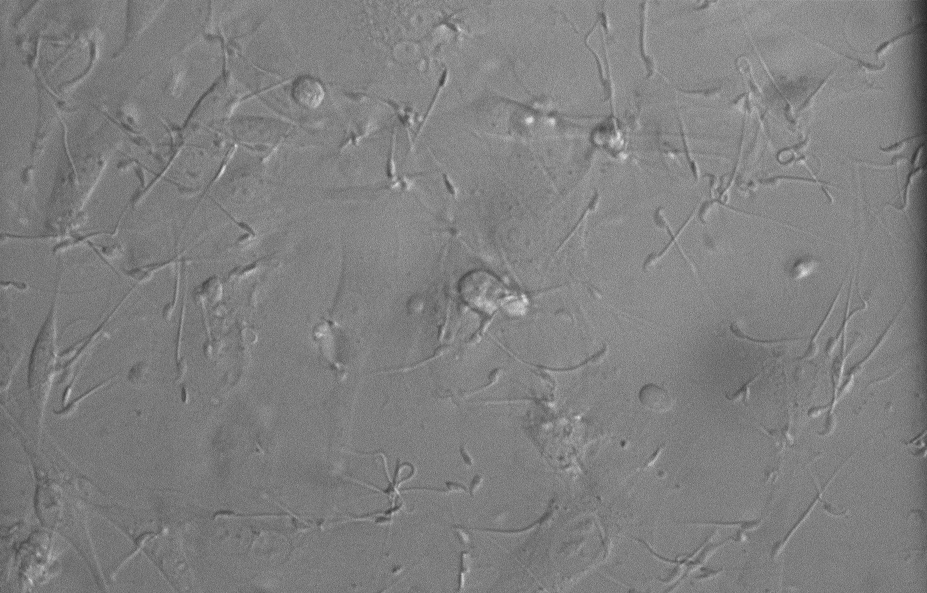

Supplement: Source data 3. [file elife-94228-data3.zip › Raw Images/Figure 1/Panel E and Movie 1/DIC/DIC_s13_t12.TIF]

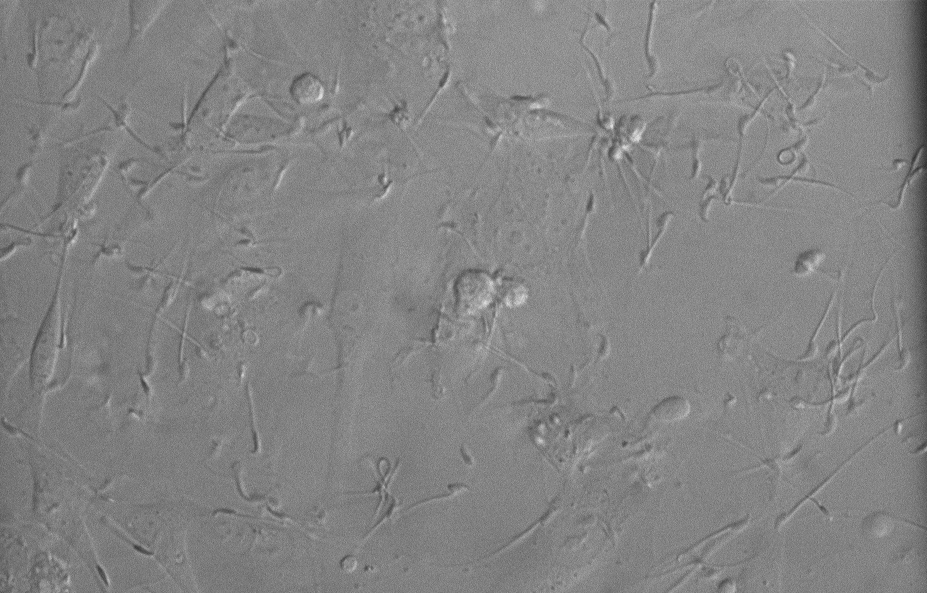

Supplement: Source data 3. [file elife-94228-data3.zip › Raw Images/Figure 1/Panel E and Movie 1/DIC/DIC_s13_t10.TIF]

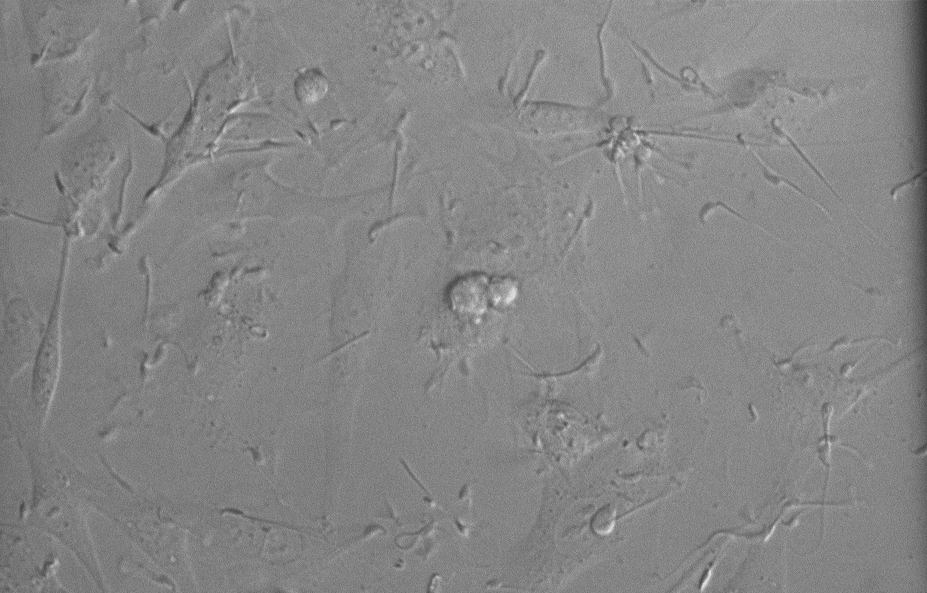

Supplement: Source data 3. [file elife-94228-data3.zip › Raw Images/Figure 1/Panel E and Movie 1/DIC/DIC_s13_t6.TIF]

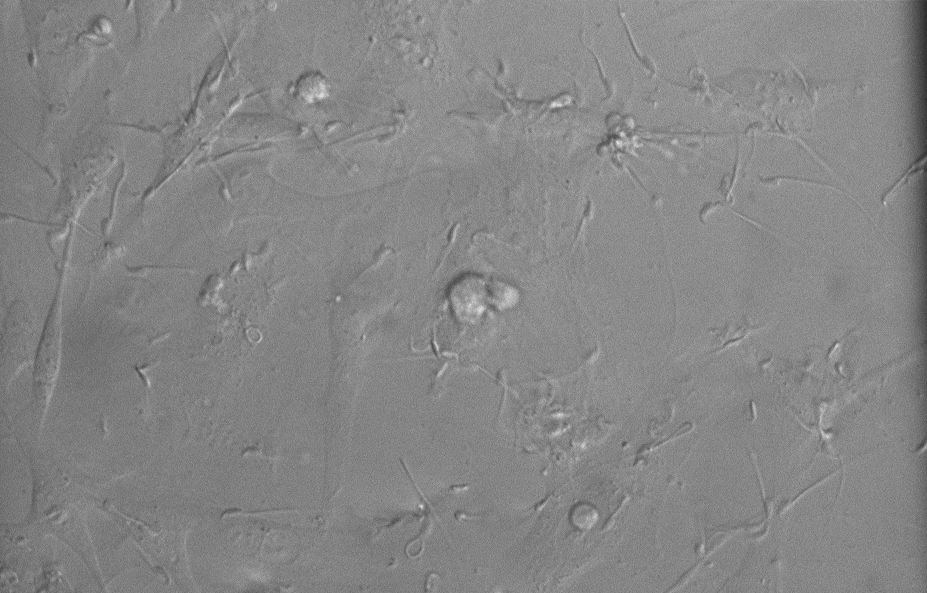

Supplement: Source data 3. [file elife-94228-data3.zip › Raw Images/Figure 1/Panel E and Movie 1/DIC/DIC_s13_t7.TIF]

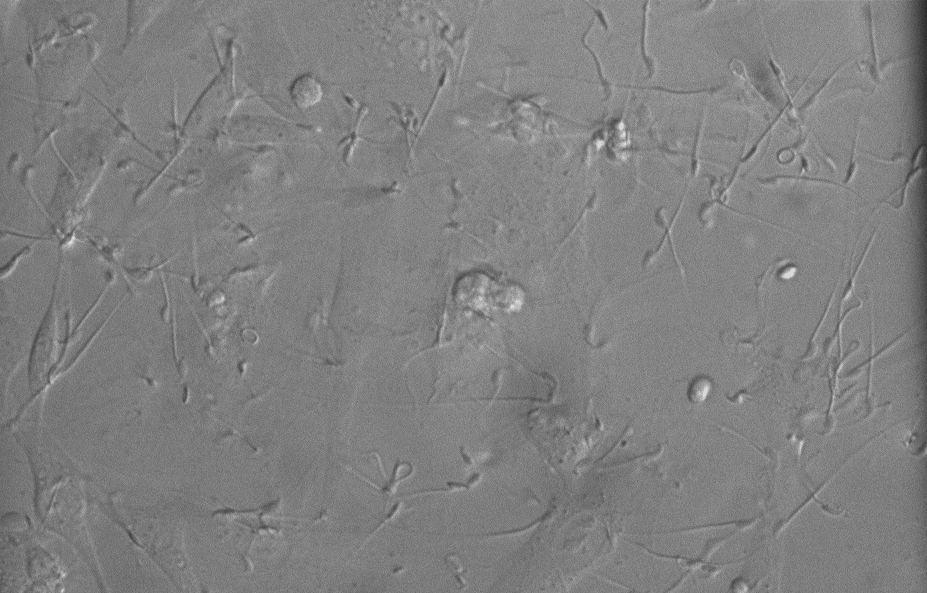

Supplement: Source data 3. [file elife-94228-data3.zip › Raw Images/Figure 1/Panel E and Movie 1/DIC/DIC_s13_t11.TIF]

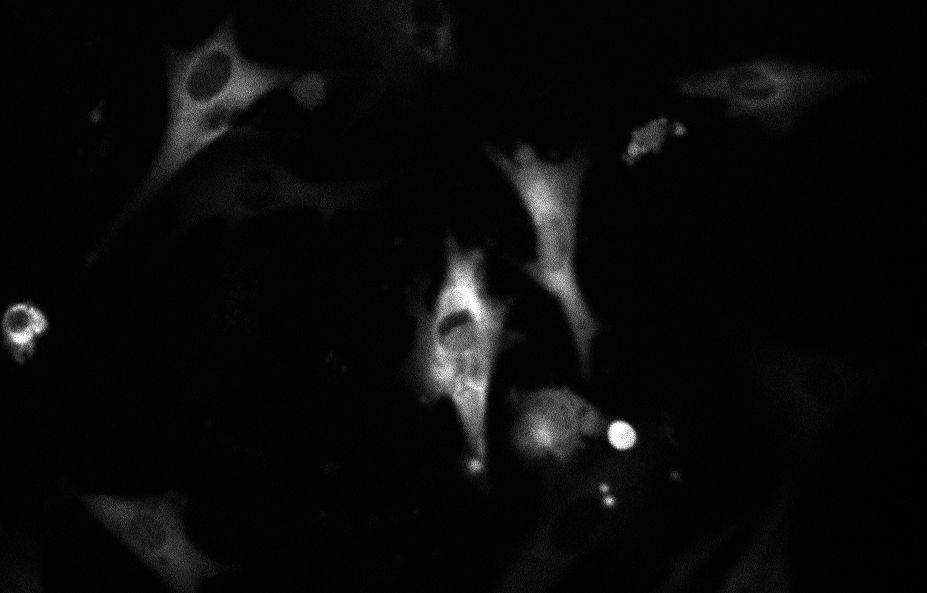

Supplement: Source data 3. [file elife-94228-data3.zip › Raw Images/Figure 1/Panel E and Movie 1/Stack GFP.tif]

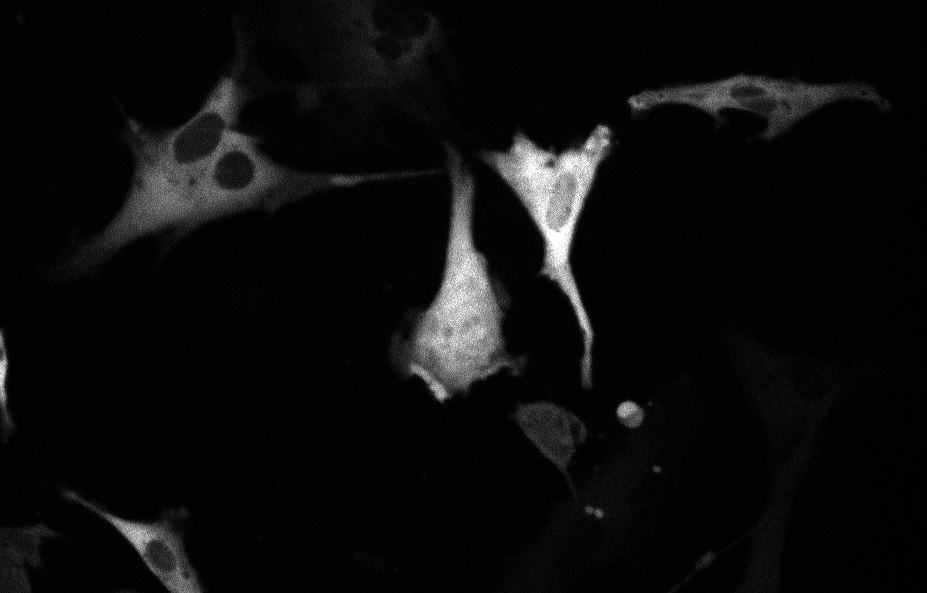

Supplement: Source data 3. [file elife-94228-data3.zip › Raw Images/Figure 1/Panel E and Movie 1/GFP/GFP_w1488nm_s13_t17.TIF]

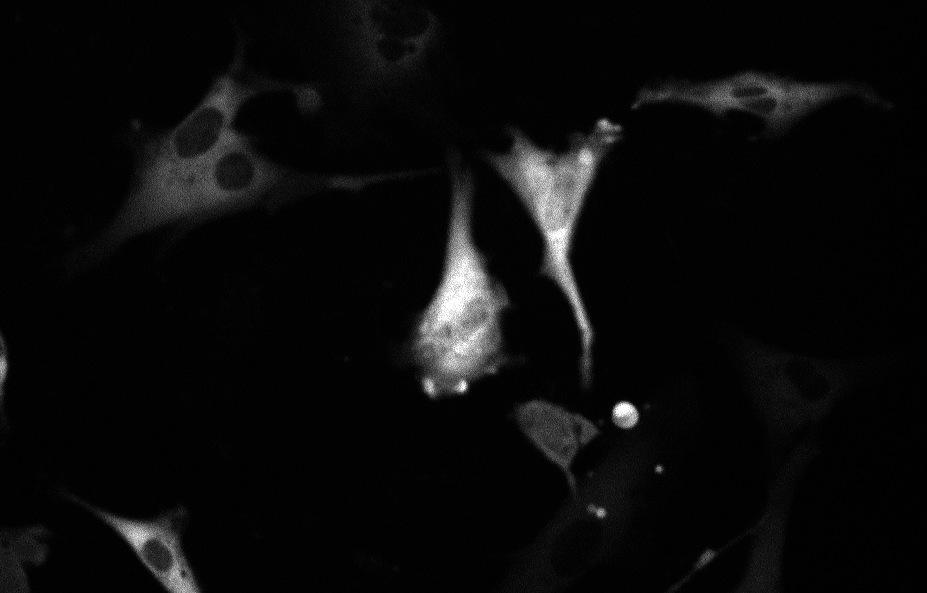

Supplement: Source data 3. [file elife-94228-data3.zip › Raw Images/Figure 1/Panel E and Movie 1/GFP/GFP_w1488nm_s13_t16.TIF]

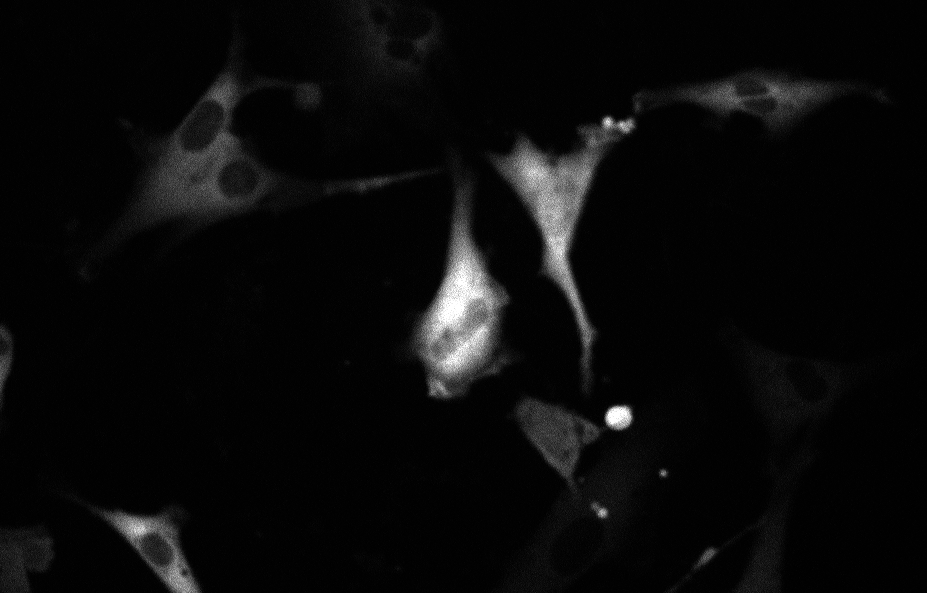

Supplement: Source data 3. [file elife-94228-data3.zip › Raw Images/Figure 1/Panel E and Movie 1/GFP/GFP_w1488nm_s13_t14.TIF]

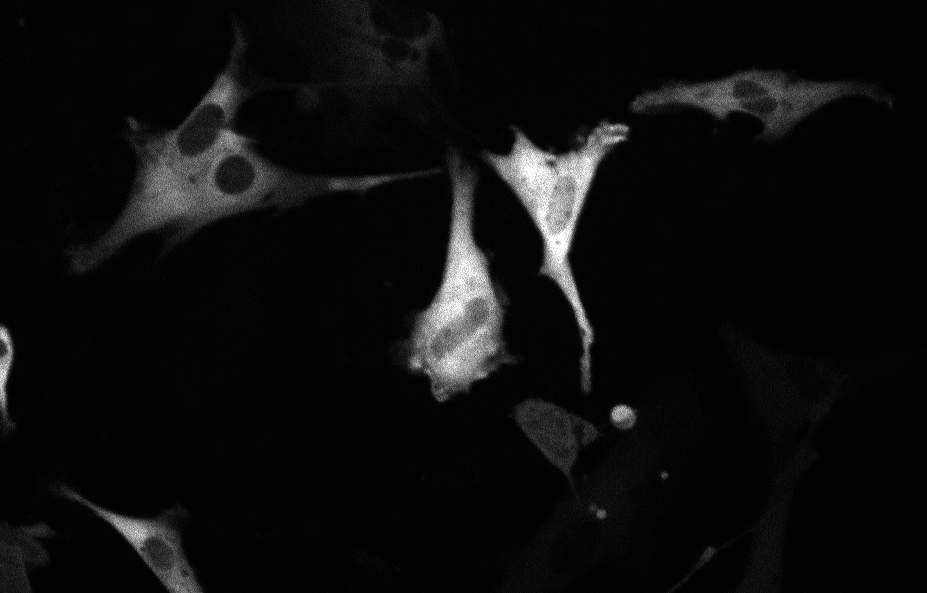

Supplement: Source data 3. [file elife-94228-data3.zip › Raw Images/Figure 1/Panel E and Movie 1/GFP/GFP_w1488nm_s13_t15.TIF]

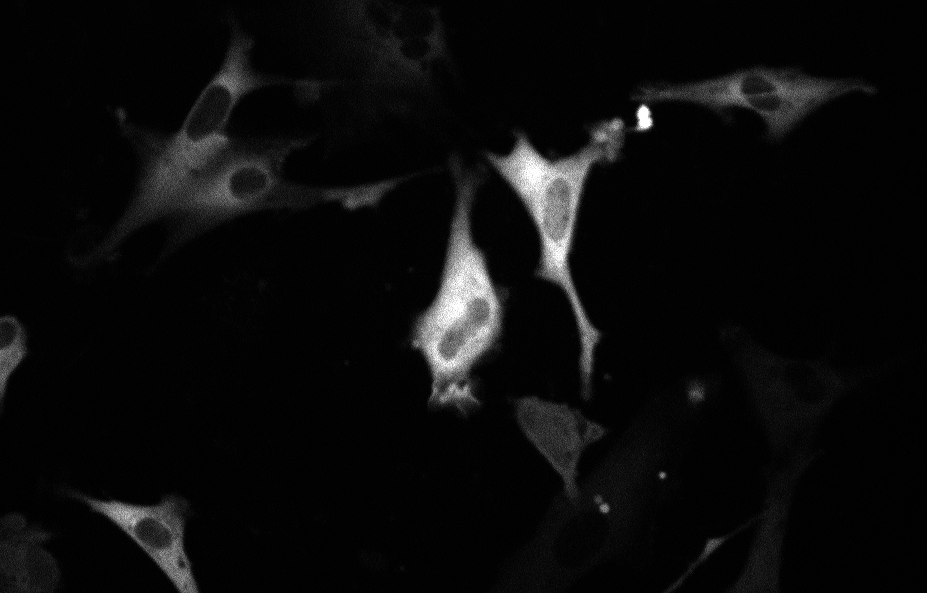

Supplement: Source data 3. [file elife-94228-data3.zip › Raw Images/Figure 1/Panel E and Movie 1/GFP/GFP_w1488nm_s13_t11.TIF]

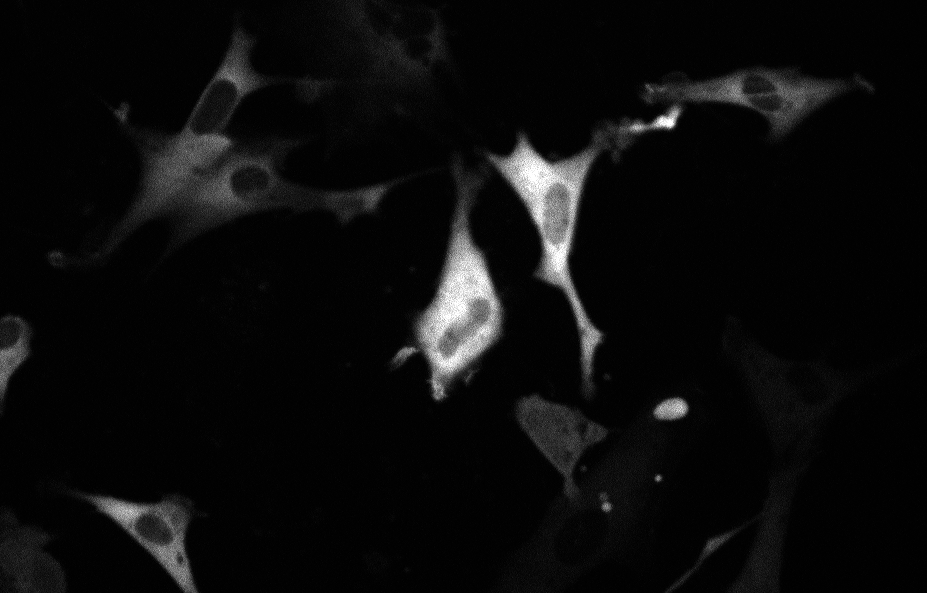

Supplement: Source data 3. [file elife-94228-data3.zip › Raw Images/Figure 1/Panel E and Movie 1/GFP/GFP_w1488nm_s13_t10.TIF]

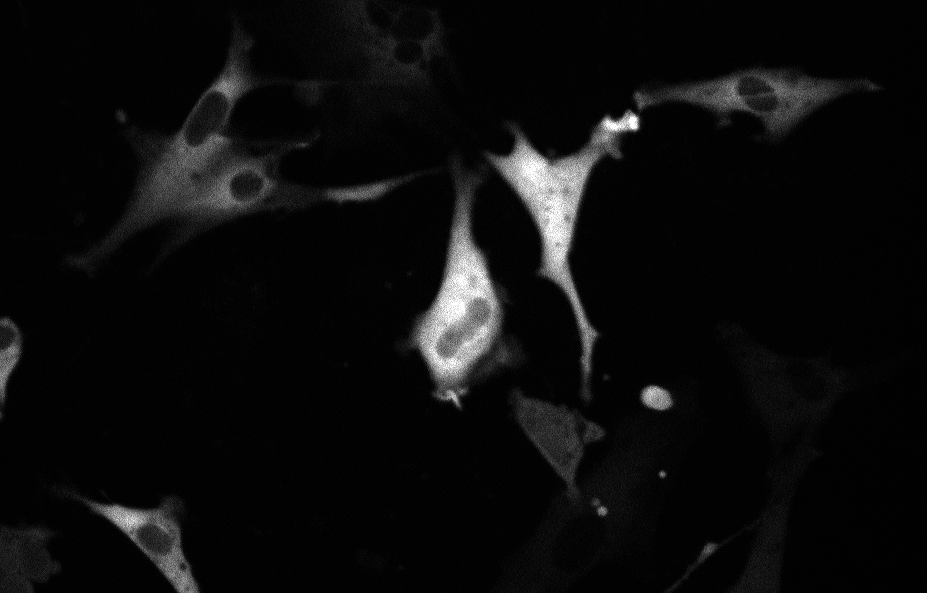

Supplement: Source data 3. [file elife-94228-data3.zip › Raw Images/Figure 1/Panel E and Movie 1/GFP/GFP_w1488nm_s13_t12.TIF]

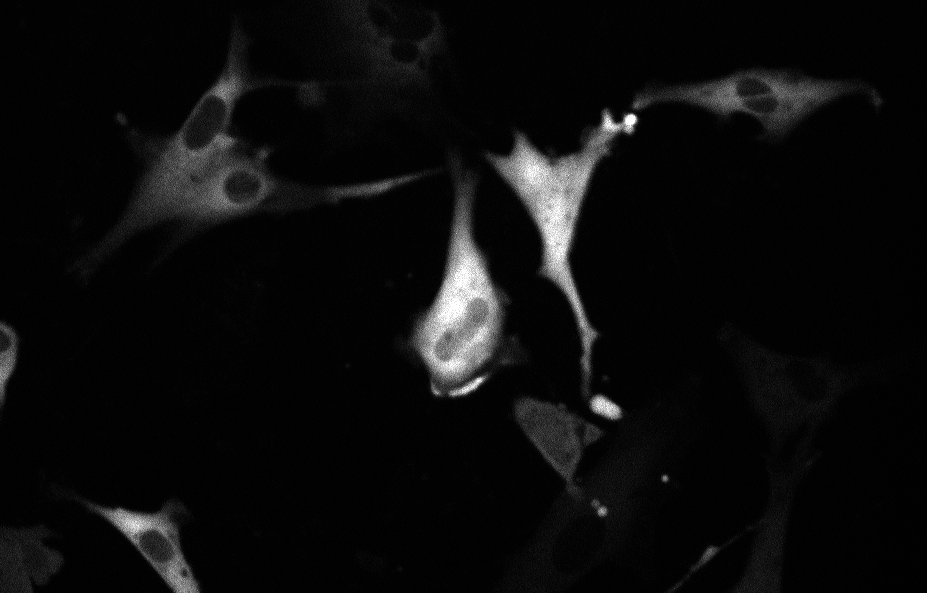

Supplement: Source data 3. [file elife-94228-data3.zip › Raw Images/Figure 1/Panel E and Movie 1/GFP/GFP_w1488nm_s13_t13.TIF]

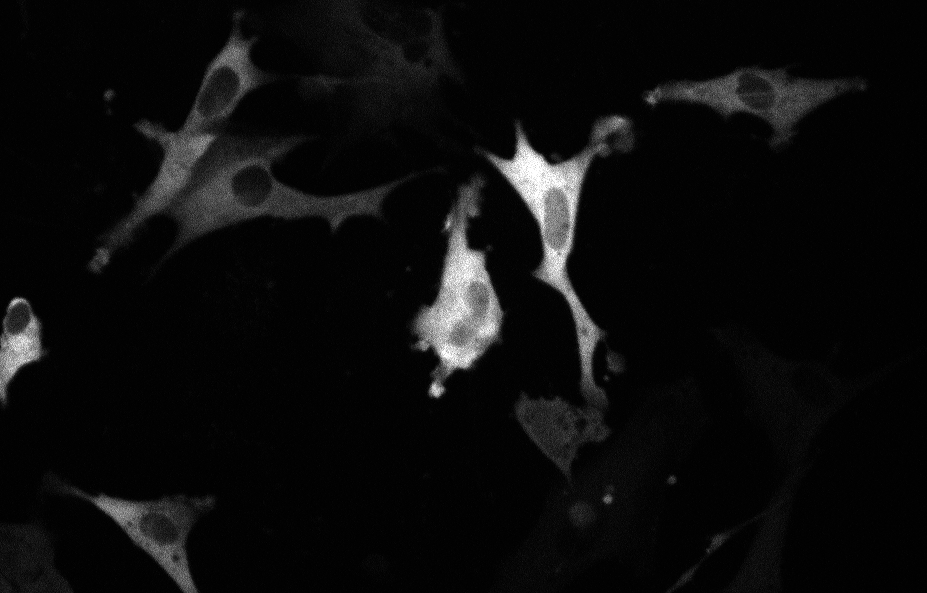

Supplement: Source data 3. [file elife-94228-data3.zip › Raw Images/Figure 1/Panel E and Movie 1/GFP/GFP_w1488nm_s13_t7.TIF]

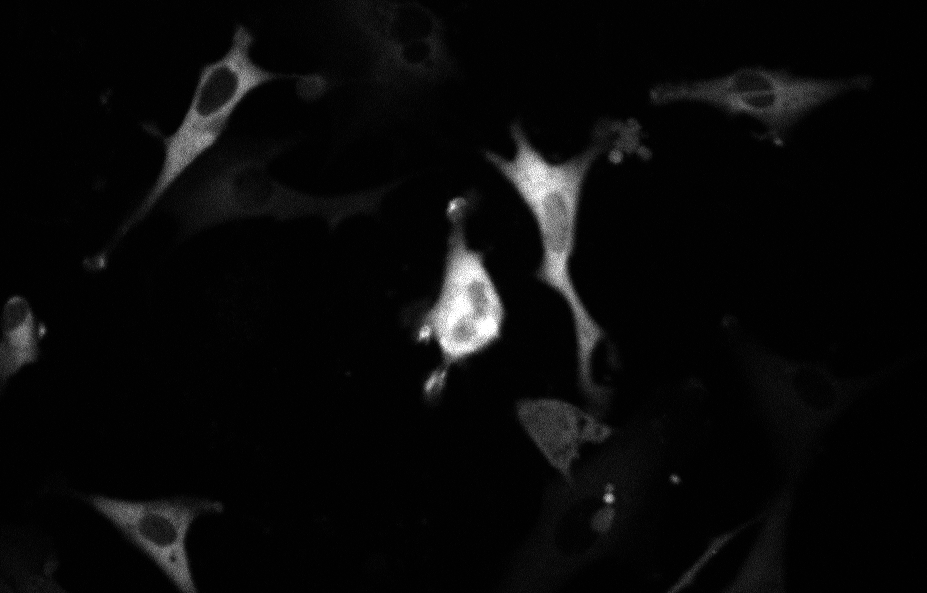

Supplement: Source data 3. [file elife-94228-data3.zip › Raw Images/Figure 1/Panel E and Movie 1/GFP/GFP_w1488nm_s13_t6.TIF]

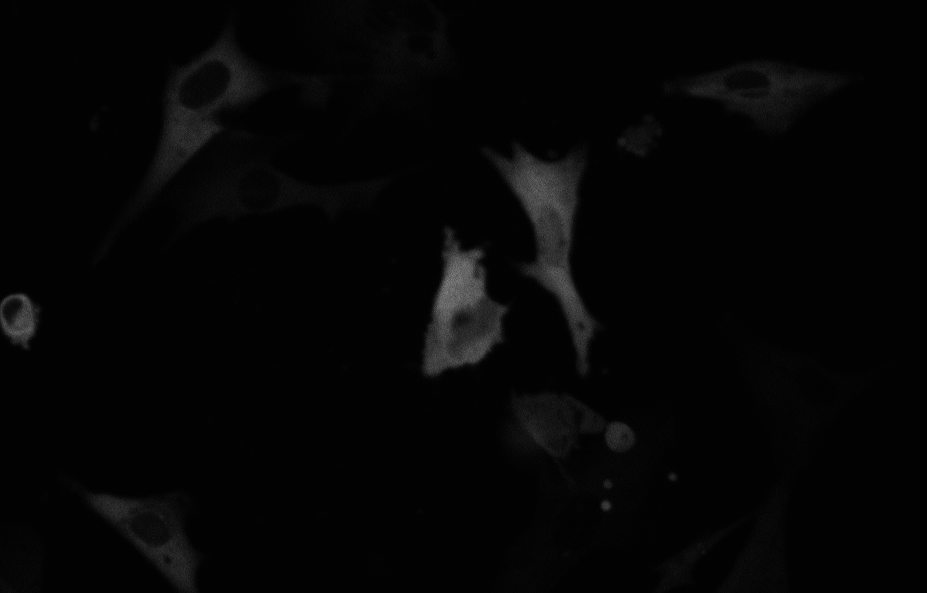

Supplement: Source data 3. [file elife-94228-data3.zip › Raw Images/Figure 1/Panel E and Movie 1/GFP/GFP_w1488nm_s13_t4.TIF]

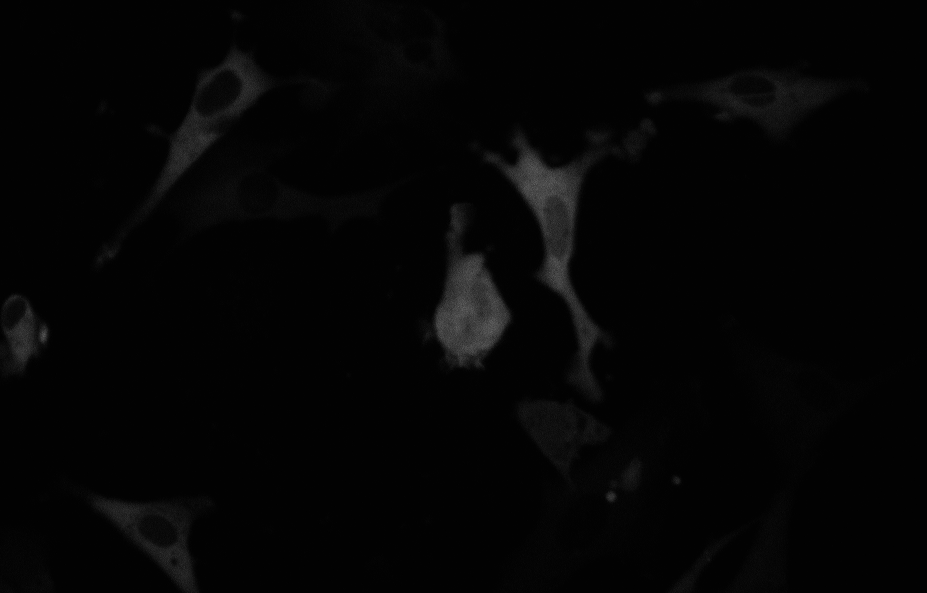

Supplement: Source data 3. [file elife-94228-data3.zip › Raw Images/Figure 1/Panel E and Movie 1/GFP/GFP_w1488nm_s13_t5.TIF]

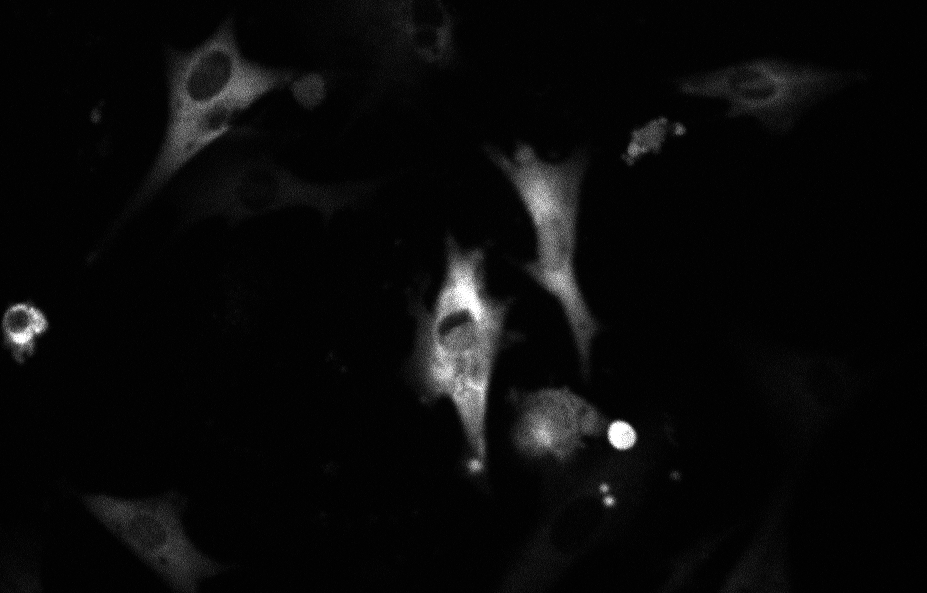

Supplement: Source data 3. [file elife-94228-data3.zip › Raw Images/Figure 1/Panel E and Movie 1/GFP/GFP_w1488nm_s13_t1.TIF]

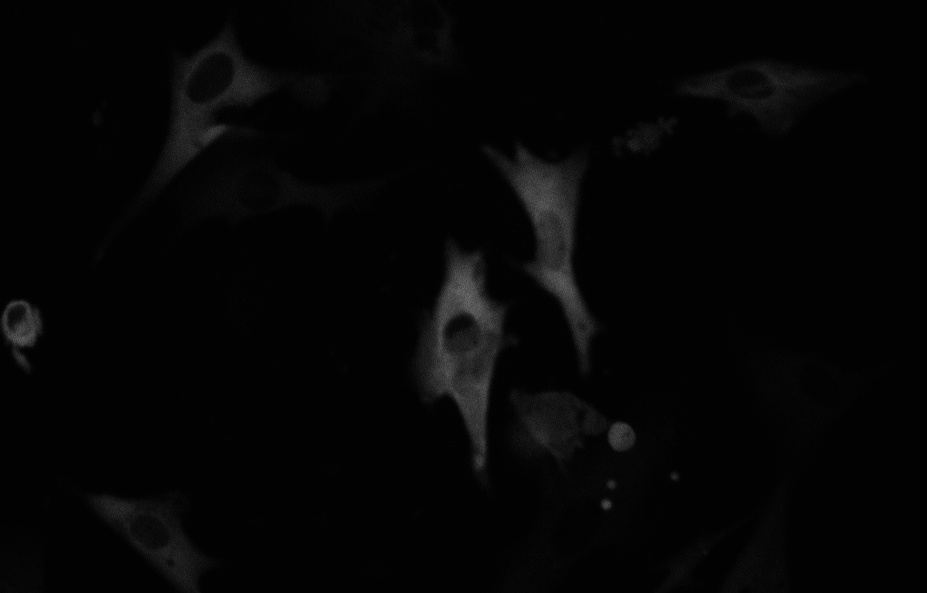

Supplement: Source data 3. [file elife-94228-data3.zip › Raw Images/Figure 1/Panel E and Movie 1/GFP/GFP_w1488nm_s13_t2.TIF]

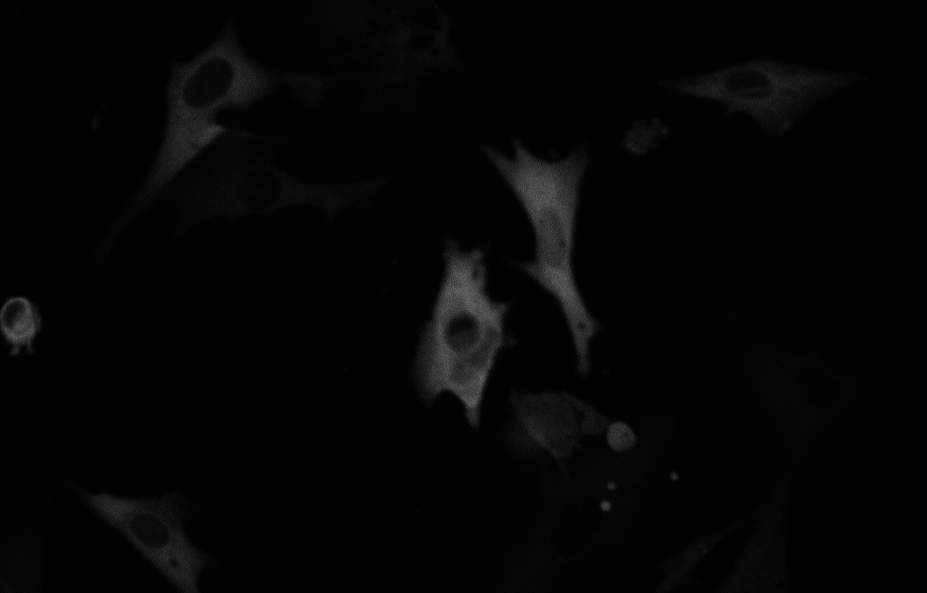

Supplement: Source data 3. [file elife-94228-data3.zip › Raw Images/Figure 1/Panel E and Movie 1/GFP/GFP_w1488nm_s13_t3.TIF]

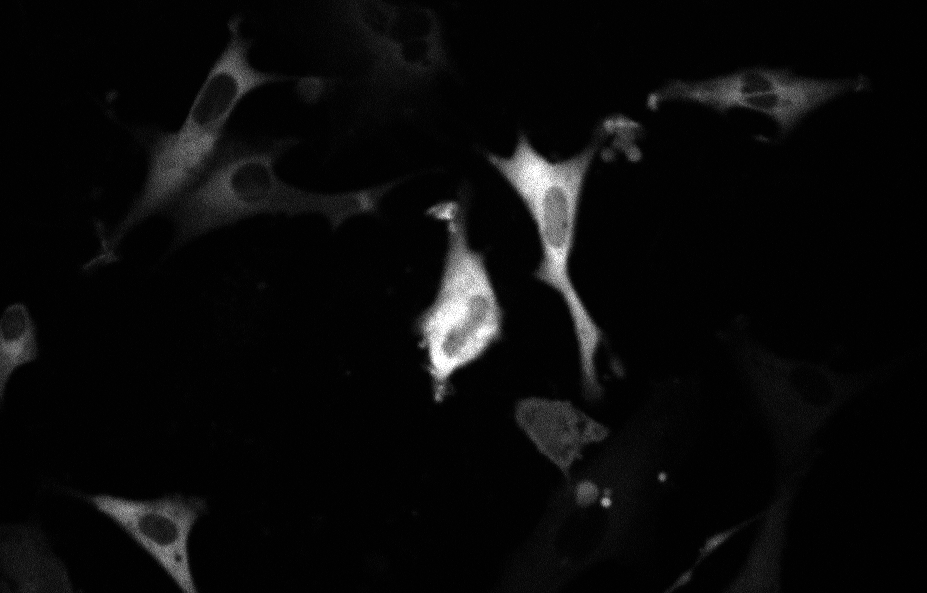

Supplement: Source data 3. [file elife-94228-data3.zip › Raw Images/Figure 1/Panel E and Movie 1/GFP/GFP_w1488nm_s13_t8.TIF]

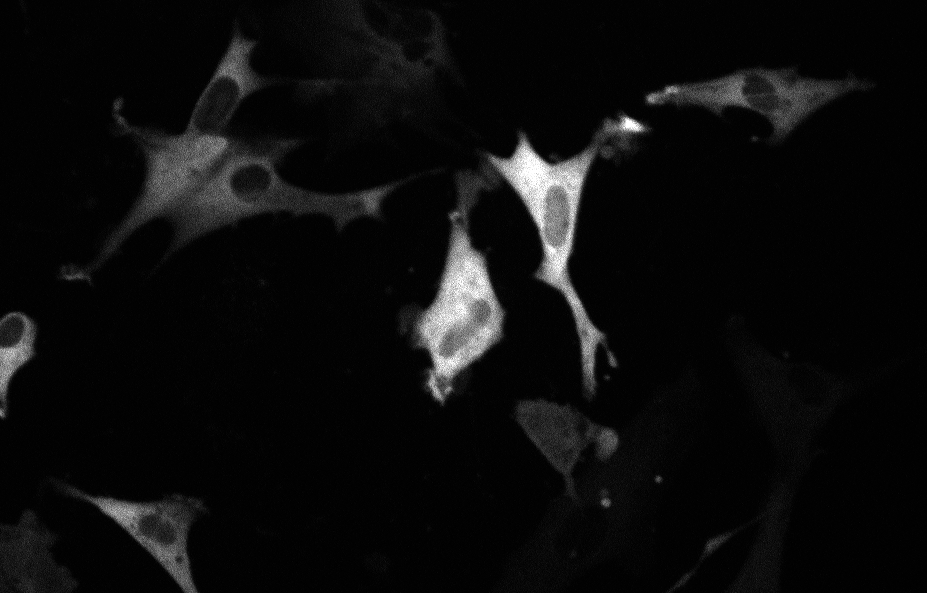

Supplement: Source data 3. [file elife-94228-data3.zip › Raw Images/Figure 1/Panel E and Movie 1/GFP/GFP_w1488nm_s13_t9.TIF]

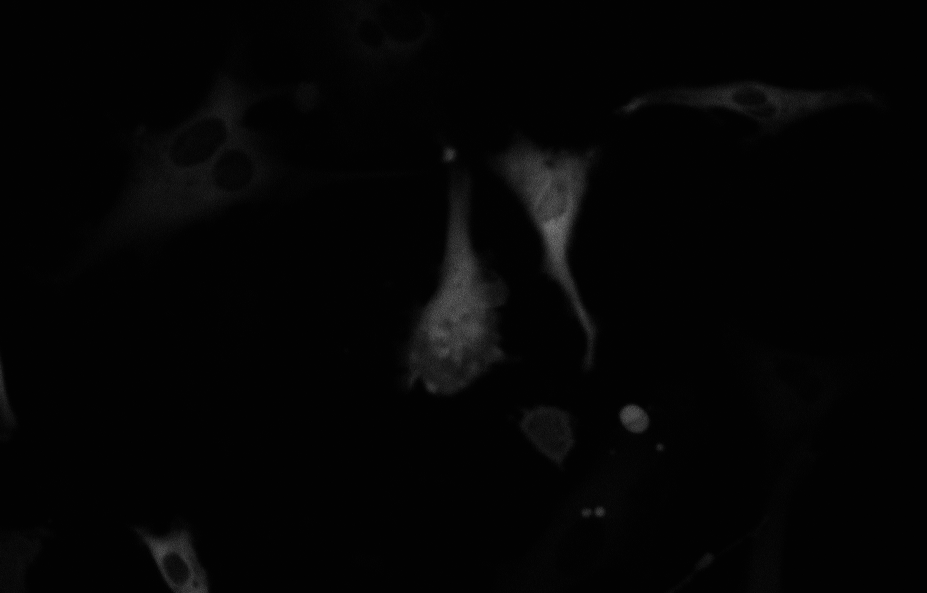

Supplement: Source data 3. [file elife-94228-data3.zip › Raw Images/Figure 1/Panel E and Movie 1/GFP/GFP_w1488nm_s13_t22.TIF]

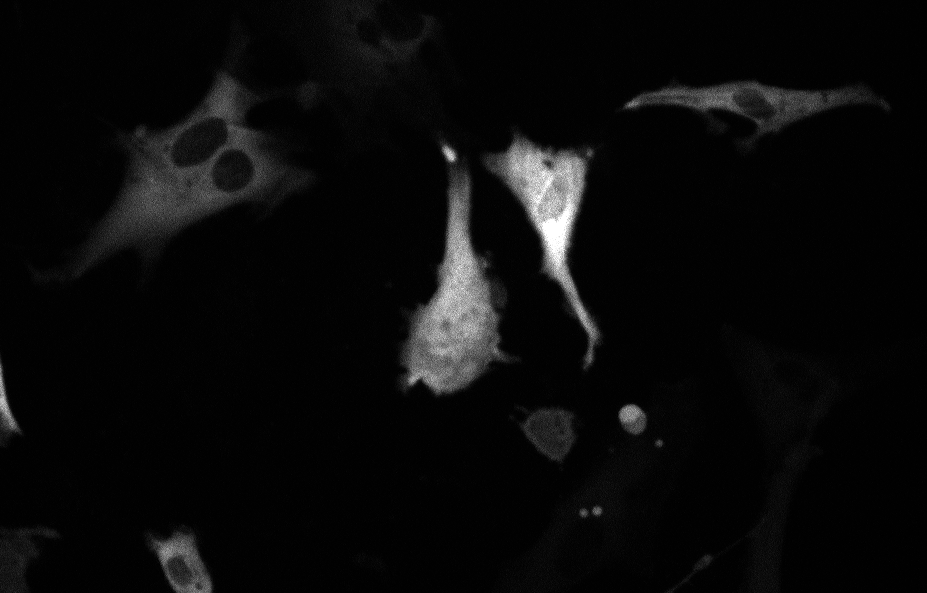

Supplement: Source data 3. [file elife-94228-data3.zip › Raw Images/Figure 1/Panel E and Movie 1/GFP/GFP_w1488nm_s13_t23.TIF]

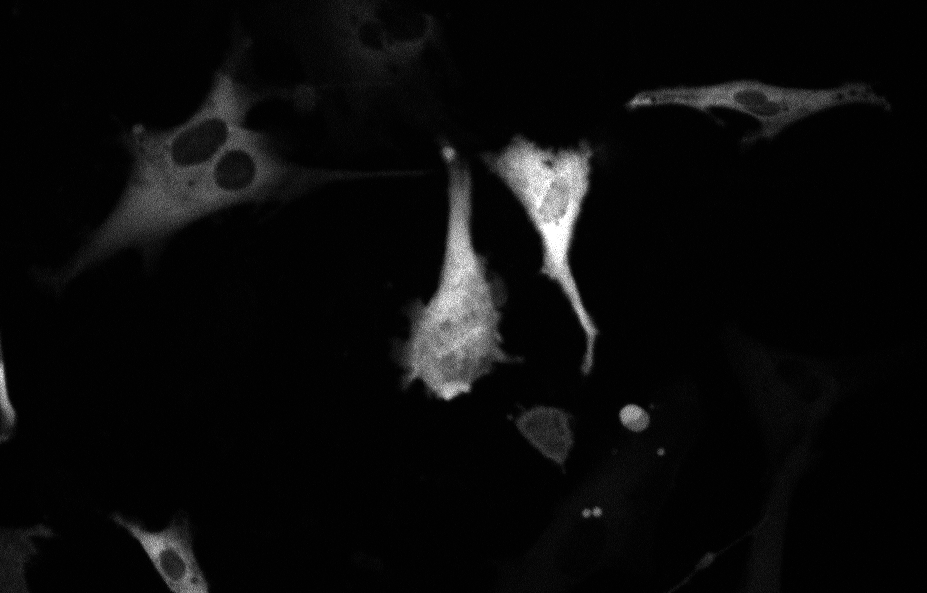

Supplement: Source data 3. [file elife-94228-data3.zip › Raw Images/Figure 1/Panel E and Movie 1/GFP/GFP_w1488nm_s13_t21.TIF]

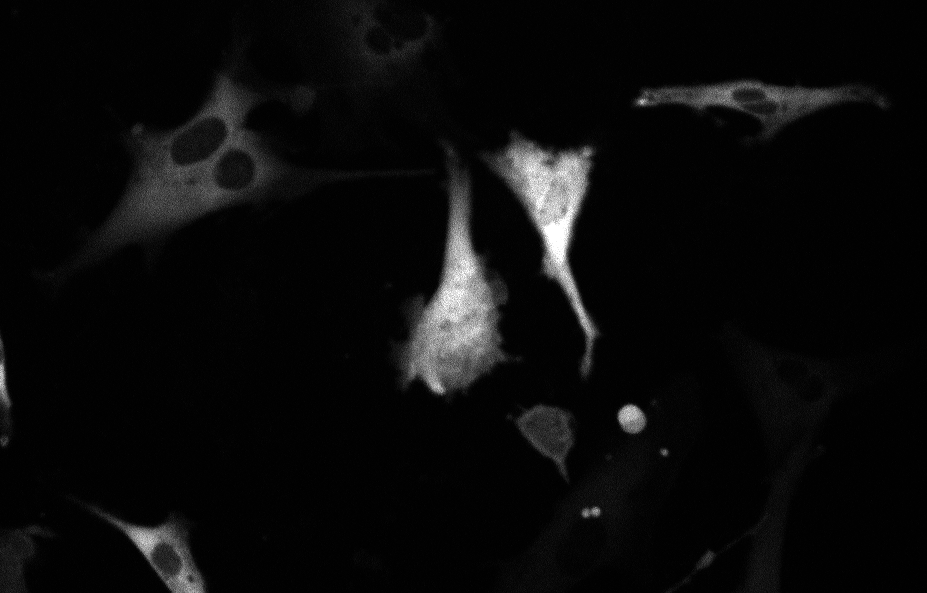

Supplement: Source data 3. [file elife-94228-data3.zip › Raw Images/Figure 1/Panel E and Movie 1/GFP/GFP_w1488nm_s13_t20.TIF]

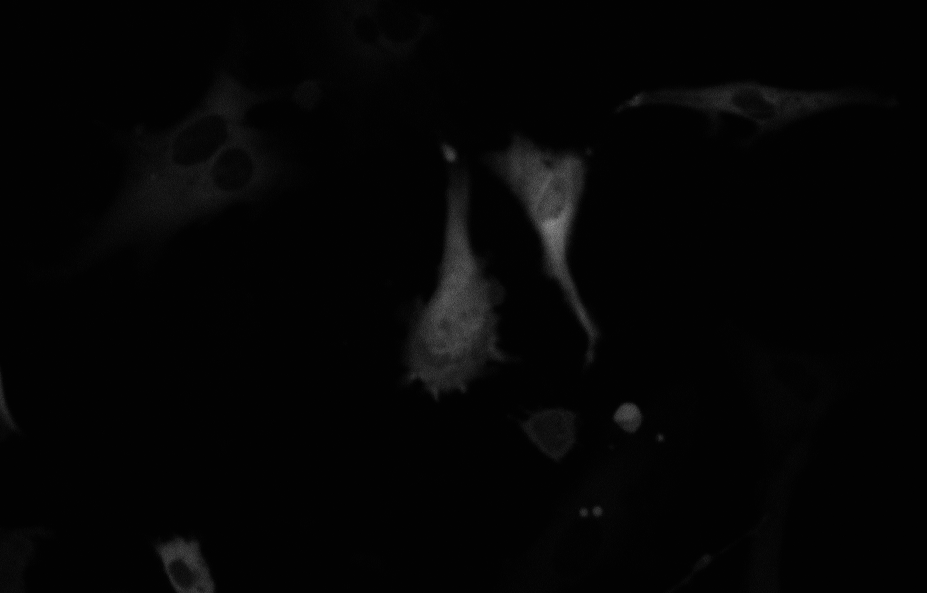

Supplement: Source data 3. [file elife-94228-data3.zip › Raw Images/Figure 1/Panel E and Movie 1/GFP/GFP_w1488nm_s13_t24.TIF]

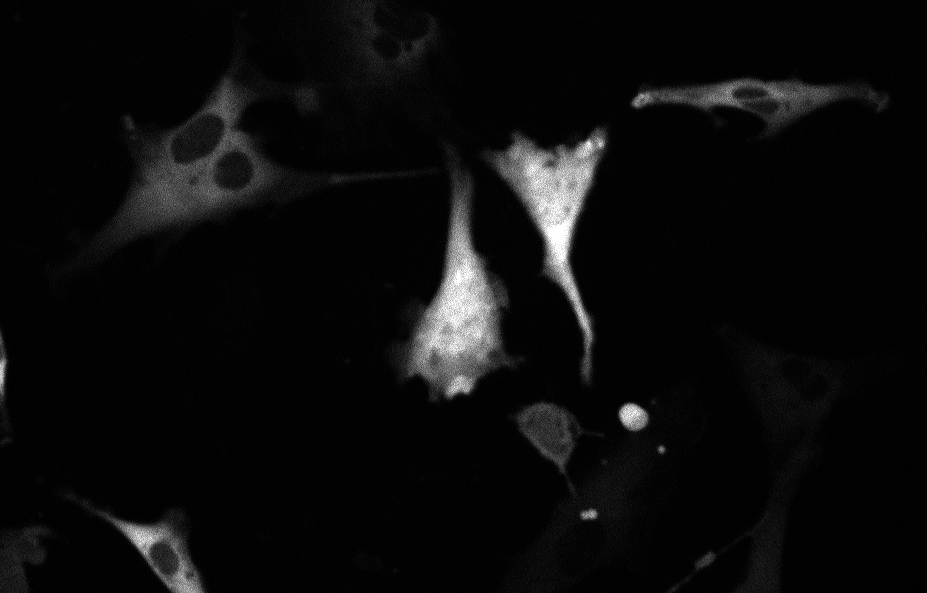

Supplement: Source data 3. [file elife-94228-data3.zip › Raw Images/Figure 1/Panel E and Movie 1/GFP/GFP_w1488nm_s13_t18.TIF]

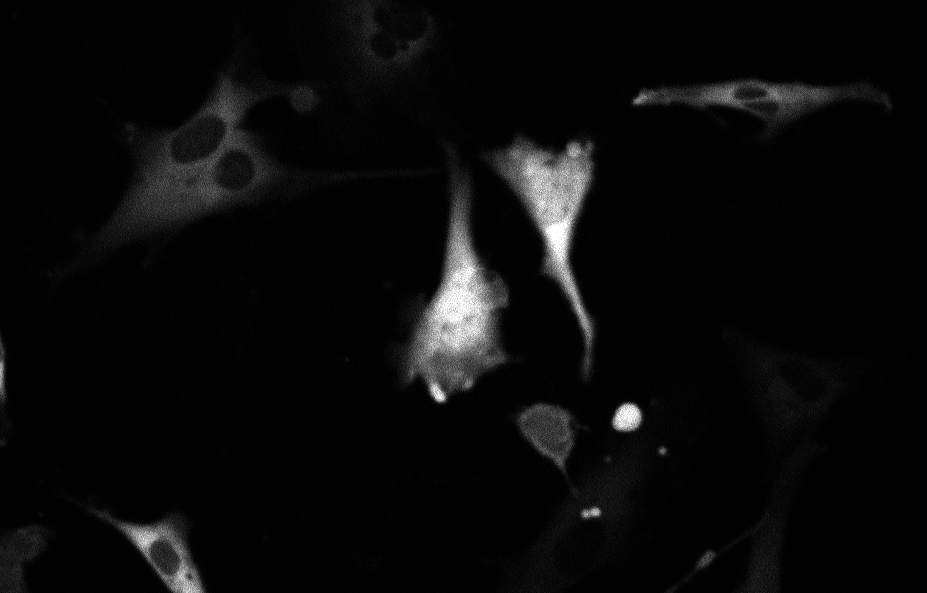

Supplement: Source data 3. [file elife-94228-data3.zip › Raw Images/Figure 1/Panel E and Movie 1/GFP/GFP_w1488nm_s13_t19.TIF]

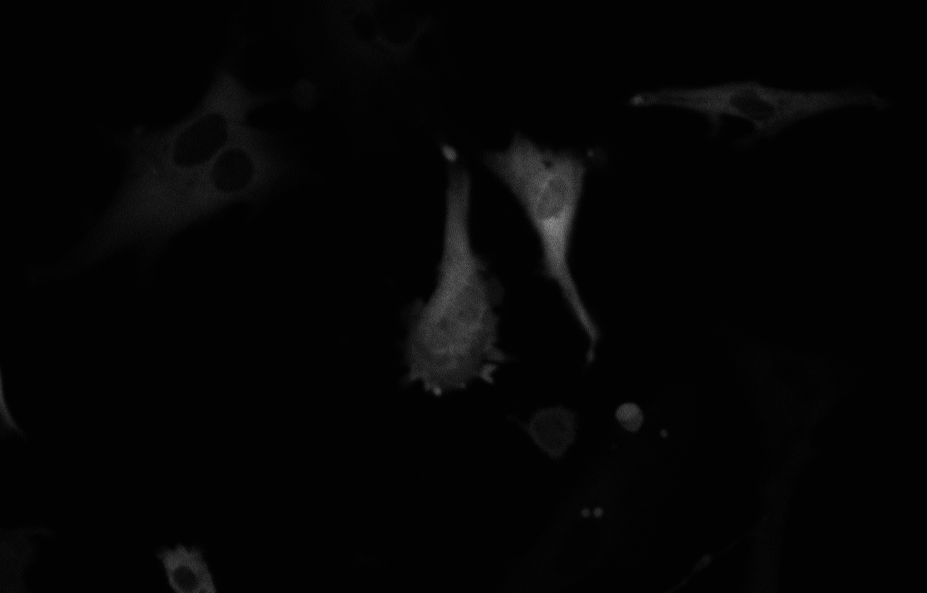

Supplement: Source data 3. [file elife-94228-data3.zip › Raw Images/Figure 1/Panel E and Movie 1/GFP/GFP_w1488nm_s13_t25.TIF]

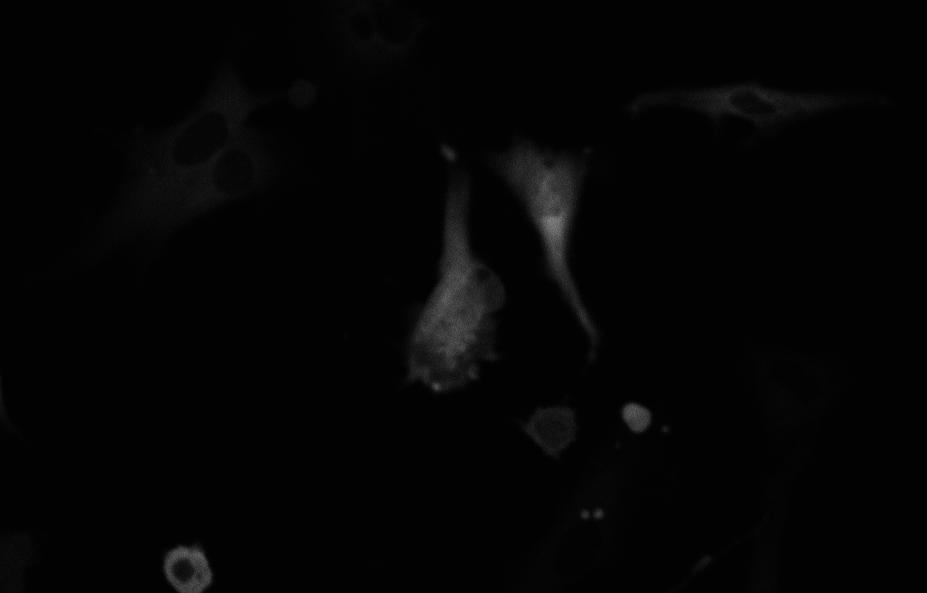

Supplement: Source data 3. [file elife-94228-data3.zip › Raw Images/Figure 1/Panel E and Movie 1/GFP/GFP_w1488nm_s13_t26.TIF]
